# Literacy as a determining factor for brain organization: from Lecours’ contribution to the present day

**DOI:** 10.1590/S1980-57642009DN20300002

**Published:** 2008

**Authors:** Maria Alice de Mattos Pimenta Parente, Rochele Paz Fonseca, Lilian Cristine Scherer

**Affiliations:** 1 Neuropsycholinguistic Laboratory, Psychology Institute, Federal University of Rio Grande do Sul, UFRGS; 2 Neuropsychology Group, Pontifícia Universidade Católica do Rio Grande do Sul, PUCRS; 3 Linguistics Faculty, Post-Graduation Program in Linguistics – Reading and Cognition, University of Santa Cruz do Sul, UNISC

**Keywords:** literacy, education, human cognition

## Abstract

This review aimed to discuss the influence of literacy and formal education on human brain organization, based on evidence drawn from three sources:

(1) results and limitations of a project coordinated by André Roch Lecours on the influence of illiteracy on brain organization and of studies on aphasia in illiterate populations;
(2) data on the impact of schooling on the neuropsychological assessment of healthy and brain-damaged individuals, and
(3) studies on the effect of schooling on dementia.

These findings suggest that schooling and literacy processes influence cerebral organization of healthy individuals, as well as of brain-lesion individuals and those with dementia. Concerning illiteracy, the systematic pioneering studies developed by Lecours and the continuity of his investigations were essential to alert the scientific and clinical communities to take into account the role of educational experience on cognitive processing and its brain substrates.

## Abstract (pt)

Esta revisão tem por objetivo discutir a influência da alfabetização e da educação formal na organização do cérebro humano, com base em evidências aportadas por três fontes:

(1) resultados e limitações de um projeto coordenado por André Roch Lecours sobre a influência do analfabetismo na organização cerebral e de estudos sobre afasia em populações analfabetas;
(2) dados sobre o impacto da escolaridade na avaliação neuropsicológica de indivíduos saudáveis e com lesão cerebral, e
(3) estudos sobre o efeito da escolaridade na demência.

Tais achados sugerem que processos de escolaridade influenciam a organização cerebral de indivíduos saudáveis, bem como a de indivíduos com lesão cerebral e com quadro de demência. Com relação ao analfabetismo, os estudos sistemáticos pioneiros de Lecours, bem como a continuidade de suas investigações, foram essenciais para alertar as comunidades científicas e clínicas quanto à necessidade de considerarem o papel da experiência educacional no processamento cognitivo e em seus substratos cerebrais.

## Introduction

In the 1980s researchers from different cities in Brazil worked together with a French-Canadian group led by Professors André Roch Lecours (1936-2005), from the University of Montreal, and Jaques Mehler, from the *École des hautes etudes in* Paris, in a broad study entitled “Literacy as a determining factor for human brain organization”. This research verified whether writing ability development and schooling affected cerebral organization. In the context of a period prior to the availability of cerebral imaging, this experimental research paradigm was composed by comparison of four groups of patients with unilateral brain lesion, with their respective controls matched for number of years of education. Clinical groups were comprised right hemisphere (RH) lesion patients and left hemisphere (LH) lesion patients, both grouped into illiterates or literates, while the two control groups were formed by neurologically healthy individuals, matched by schooling level, age and gender. The aim was to investigate the effect of formal learning of writing. Accordingly, the illiterate group in cluded only individuals who, besides being unable to read or write, had never attended classes at school.

Two results were evident and generated further repercussions. The first finding related to the influence of schooling on simple tests from aphasia evaluation instruments^1^, while the second finding concerned RH participation in illiterate populations while solving naming tasks.^2,3^

This review aimed to discuss the influence of literacy and formal education on human brain organization, based on evidence drawn from three sources:

(1) results and limitations of the project mentioned above, coordinated by Lecours, and of studies on aphasia in illiterate populations;
(2) data on the impact of schooling on the neuropsychological assessment of healthy and brain-damaged individuals, and
(3) studies on the effect of education on dementia.

To accomplish this aim, the historical and methodological context will first be outlined followed by the results of Lecours’ research, Subsequently, a review of some topics will be presented, which after more than 25 years have complemented the discussions, along with the limitations of research into the influence of literacy on human brain organization.

Currently, there are a large number of neuropsychological studies focusing on the influence of sociocultural factors and, particularly schooling level.^4,5^ This discussion goes beyond the impact of neuropsychological assessment and beyond focal lesions. A relevant issue concerns the extent to which schooling can be associated to higher cognitive quality in aging.

### Studies developed by Lecours and colleagues and research on aphasia in illiterate populations

The relationship between illiteracy and aphasia is permeated by the notion of LH dominance for language. In this context, it has been hypothesized that schooling and written language learning may influence and modify this dominance.^6,7^ Evidence to confirm this hypothesis comes from the more frequent occurrence of aphasia in illiterate individuals with RH damage, associated with better spontaneous recovery of illiterate patients with LH lesion. Such observations suggest a higher importance of the RH for linguistic processing in illiterate individuals compared to literates.

After these pioneering studies, several reports of non-systematic cases of illiterate aphasics have followed, highlighting the absence or rare occurrence of linguistic impairment after LH lesion, associated to crossed aphasia in RH lesions.^8-10^ In a more systematic way, three studies have investigated groups of patients with varying education levels, aiming to characterize hemispheric lateralization for language in illiterate individuals. Regarding the first study, 62 right-handed and 3 left-handed adults, all LH brain-damaged, were grouped according to schooling:11 37 were classified as illiterate, with an average of 2.5 years of schooling; 14, semiliterate, with an average of 5.6 years of schooling; and 14 literate, with 10.6 years of formal education. In descending order, greatest transitory or persistent aphasia was present in literate adults (78%), followed by semiliterate adults (64%), and finally illiterate adults (36%). Since there were significant differences in the comparison between the performance of the first and third groups, the authors concluded that literacy exerts influence on the cerebral dominance for language.

On the other hand, incongruent results have been found in the literature. For instance, a Portuguese study (second study) with a significant sample size investigated 209 schooled adults and 38 illiterate adults^12^. Regarding the proportion of aphasia occurrences, similarities were seen between the two groups. Among schooled participants, language impairments were detected in 114 patients (54.5%), while among illiterates this pattern was observed in 21 (55.2%). There was only one illiterate patient with a RH lesion. The other illiterate patients, akin to schooled participants, had LH lesions. In contrast to the previous study, the role of the RH in language dominance did not evidence any changes.

In a bid to achieve greater systematization of studies on illiteracy, an investigation coordinated by Lecours and colleagues (third study) based on a more tightly-controlled experimental design was developed in Brazil^2^: besides two control groups, one for each level of schooling, patients with exclusive LH or RH damage were selected. A total of 296 adults (153 males and 143 females), all monolingual and Brazilian Portuguese native speakers, participated in the study. Considering the distribution of the groups of different schooling levels by lesion side, there were 48 illiterate participants (unable to read or write, with no school attendance) and 61 literate participants (8 or more years of formal schooling) presenting LH damage, and 47 illiterates and 32 literate participants with RH lesions. Neuropsychological language assessment in this study encompassed simple tests of naming, repetition and oral comprehension. Brain-damaged patients scored below controls across all tasks. In repetition and comprehension tasks, this difference was statistically significant for patients with LH lesion, but not for RH brain-damaged patients. This finding was expected, indicating LH post-lesion aphasia. In naming tasks, however, this statistically significant difference between clinic and control group was observed among illiterate patients with RH lesion, as well as literate and illiterate LH brain-damaged patients. Consequently, the authors proposed that their findings point to a higher participation of the RH in illiterate populations in language processing than in literate populations. This hypothesis suggests that cerebral language representation seems to be more bilateral in illiterate individuals than in schooled individuals, although “cerebral dominance” continues the norm for both groups.

Despite being pioneering in terms of thematic selection and methodological rigor, two limitations have been detected in the project coordinated by Lecours. The first concerns the reduced specification of cerebral substrate, overcome by the use of positron emission topography (PET) in illiterate individuals^13,14^. The second limitation relates to the impossibility of dissociating the influence of illiteracy from the effect of formal schooling, in which several other cognitive processes are developed besides written language.

The findings produced by Lecours’ group, reported above, have recently been criticized.^15^ The authors of this criticism claimed that the use of graphical drawings in neuropsychological evaluation is more difficult in non-schooled individuals and, as a consequence, results did not demonstrate lesion effects but instead reflected pre-morbid differences in comparing illiterate to literate individuals. However, careful reading of the two articles written by Lecours and colleagues^1,2^ shows that difficulties related to schooling were controlled for in non-brain-damaged participants in the first study.^1^ Results from RH and LH brain-damaged literate and illiterate patients were always obtained by comparison to data from non-brain-damaged controls.^2^ Thus, the comparison between literate and illiterate patients was developed by an analysis of the differences between literate controls and literate patients with RH lesion, and between illiterate controls and illiterate patients with lesion in this hemisphere. This safeguard in terms of methodology was intended to offset pre-morbid effects, emphasizing RH influence on illiterate participants’ language processing, identified in research using PET and in Reis and Peterson’s study.^15^

A follow-up procedure for Lecours’ project entailed a reassessment carried our six months after the initial evaluation. The authors had the opportunity to examine 59 patients with unilateral brain damage from the fist study who had not been submitted to a speech therapy intervention.^3^ This group was composed of 18 illiterate and 21 literate patients with LH lesion, as well as 13 illiterate and 7 literate RH brain-damaged patients. Differences between the two evaluations were found only in data from the naming tasks. LH brain-damaged illiterate patients showed significant evolution. However, LH brain-damaged literate participants and RH brain-damaged illiterate patients did not present significant spontaneous evolution. The small number of patients in each group and the criteria based on clinical assessment did not allow analyses of the effect of lesion size and site. Notwithstanding, the test-retest method reinforces the notion that schooling and literacy influence cerebral organization for language and reiterates the higher RH participation in language processing by illiterates.

A recent study on aphasia recovery conducted in the United States observed similar recovery in a comparison between participants with low and high educational level^16^. The lack of specificity regarding the analyzed population precludes comparison with other studies. Since a socioeconomic profile scale (Holingshead) was used in the present study, the groups may not reflect the contrast present in Lecours’ study, which compared illiterate participants with no schooling to literate participants who had been exposed to formal education for more than five years.

Research with PET and statistical analysis of parametric mapping (SPM) has reported differences in brain images of Portuguese illiterate and literate elderly women in tests of non-word repetition.^17^ In the comparison between real and non-words as baseline, both groups generated similar brain images. Conversely, when non-words were analyzed taking real words as baseline, the literate group showed a wide activation area in left and right anterior insula, right fronto-opercular cortex, anterior cortex of cingulate gyrus, left basal ganglia, anterior thalamus, hypothalamus and medial part of the cerebellum. Yet in the illiterate elderly group, activation was restricted to the medial frontal region. These results reinforce the findings of increased difficulty in repeating non-words, reflected by the illiterate sample who participated in the experiment. The authors interpret these data as indicating that differences in experience, in this case, language learning, may lead to changes in neuropsycholinguistic processing. The process of becoming literate requires phonologic attention training which conducts the organization and production of motor sequences. In this way, language becomes conscious, involving the declarative memory system and not only automatic processes, which require the implicit memory system. Consequently, brain regions responsible for the cognitive functions acquired during formal writing system acquisition tend to be more associated in literate than in illiterate individuals.

Word repetition tasks involve working memory resources, and there is a general assumption that schooling and literacy have a strong effect on different types of memory processes. The role of the left prefrontal cortex (PFC) and medial temporal lobe (MTL) in encoding auditory-verbal information was studied in an event-related fMRI study comparing literate and illiterate women.^18^ For both literate and illiterate subjects, brain activation patterns demonstrated a positive correlation between task performance and activation in the left inferior PFC and MTL regions. Moreover, in this study, activations were recorded in both right and left MTL in illiterate participants. The authors suggested that illiterate subjects may have used a different strategy. Instead of relying on phonological encoding they may have used visuospatial imagery to memorize frequent short words presented in the stimuli. An alternative interpretation was based on the occurrence of a greater bilateral recruitment of brain regions among the illiterate population, in accordance with findings of language studies.

On the other hand, while investigating areas surrounding the corpus callosum, the same research team did not find any significant differences.^19^ In the anterior part of the posterior third of the *corpus callosum*, thinner in unschooled elderly ladies, fibers connect the two post-central cortical regions of each hemisphere, crucial areas for writing processing due to their involvement with visual-auditory-motor associations. The researchers maintained the assumption that the reduction in posterior areas of the *corpus callosum* in illiterate elderly women occurs due to the lack or limitation of environmental stimuli important for the development of a given ability.

To sum up, despite their limited number, studies in illiterate populations have shown that literacy has an effect on cerebral functional dynamics. Moreover, considering that schooling is a social factor dependent on experience, the investigations yielded evidence demonstrating that the brain is an organic structure which interacts closely with its environment.

### The impact of schooling on neuropsychological assessment of brain-damaged and non-brain-damaged individuals

#### Studies with non-brain-damaged individuals

Schooling is undoubtedly the most-investigated factor in the neurologically healthy populations in research focusing on normalization or in comparative analysis between groups of different levels of education. Although Lecours’ studies have demonstrated the relevance of this factor in contrasting groups, more recent investigations have highlighted this importance even in populations with diverse levels of high education.^20^ Schooling influence has been analyzed in neuropsychological evaluation of executive functions, attention,^21^ perception,^22^ memory, constructional praxias,^21^ language^23,24^ and communication.^25^ Results of the majority of studies suggest association between better performance and higher education levels, whereby schooling effect proved more evident, frequent and significant than the effect of aging.

Two categories can be depicted in the investigations of schooling in neuropsychological evaluation:

1) studies comparing the performance of groups with different schooling levels and
2) research searching for norms based on performance, according to a distribution of normative groups by schooling.

Besides the traditional comparison between literate and illiterate individuals, where the extreme educational levels highlight the difference in cognitive performance, studies which search for norms of performance according to education levels have become ever more frequent. For instance, the impact of schooling on performance in the Mini-Mental State Examination has been verified by Brazilian researchers.^26^ They found significant differences in the comparison of four education groups according to the number of years of formal schooling: illiterates and low schooling (1 to 4 incomplete years), medium schooling (4 to 8 incomplete years) and high schooling (8 years or more), yet not between low and medium levels. Similarly, Mexican researchers^21^ carried out research for the normalization of a neuropsychological battery according to age and schooling:

1) 0 years,
2) 1-4 years,
3) 5-9 years and
4) 10 years or more of schooling.

Performance in some tasks demonstrated a schooling effect (copy of semi-complex figure, language comprehension and phonological verbal fluency), while performance in other tasks was minimally influenced by this socio-demographic variable (praxias and recognition). Further, a ceiling effect was observed in the group with 10 to 24 years of schooling.

Another study corroborated the existence of a correlation between semantic memory in elderly and their schooling level.^27^ By analyzing naming and semantic memory ability of 121 elderly individuals - all submitted to the Boston Naming Test (BNT), 79 submitted to the Semantic Fluency Test (Sem-Flu) and 72 to the Pyramids and Palm Trees Test - the researchers verified a significant influence of the participants’ education level on all tests.

In this context, an important aspect to be emphasized is the complex effect of the schooling variable in interaction with age, which has been reported in the literature, while also reflecting a tendency toward reduced education effect as age progresses. In a sociodemographic investigation,^28^ findings suggested a tendency toward a reduced effect of the schooling variable in the elderly population’s performance in tasks of communicative processing assessment. This decrease in schooling influence as age progresses has also been reported in a study on performance in episodic memory tasks.^29^ Generally, it is not possible to state that the interaction between age and education is characterized as being linear. On the contrary, a relative heterogeneity in the relationship between these two variables has been observed in cases which have registered an inversion in these effects – for instance, when elderly participants with a low schooling level score higher than those with high education level.^28^ This diversity of interaction patterns between age and schooling depending on the cognitive domain has been highlighted by some authors,^21,23^ the heterogeneity in communicative performance being even higher in the elderly population.^30,31,32^

This heterogeneity is more evident in low schooling elderly individuals. For instance, among the group of adults above 65 years’ old who studied for 1 to 4 years, illiterate and semi-illiterate or functionally illiterate individuals may be found,^33^ as well as elderly adults with a high performance (Fonseca et al., in press). Factors such as varying levels of daily cognitive stimulation, linked, for instance, to the frequency and quality of reading and writing habits, may influence elderly participant performance.

Such specificities of the cognitive processing of illiterate individuals justify the development of specific assessment instruments for use in this population (for example, alternative versions of the Stroop Color-Word Test).^33^ Thus, neuropsychological evaluation practices among illiterate subjects tend to be differentiated, predominantly employing numbers as opposed to verbal written information.

#### Studies in brain-damaged individuals: the controversy over schooling influence

Results from studies involving non-brain-damaged individuals have corroborated the idea of the determining interference of schooling on participants’ performance in neuropsychological tasks. This consensus is not shared by investigations with brain-damaged patients, the findings of which tend to be more controversial. In a study on 30 LH brain-damaged patients (not aphasics), 30 RH brain-damaged patients and 30 controls,^34^ cluster analysis identified three groups according to participants’ performance on a free verbal fluency test: high performance (1), intermediate performance (2) and inferior to average performance (3). The homogeneity in brain-damaged participants’ distribution in the three clusters led to the conclusion that lesions had not been the main factor in determining group formation, but rather education level. The most schooled brain-damaged participants were concentrated in the cluster with high performance, while least schooled participants constituted the cluster with the worst performance. However, correlation between lesion impact and education was detected in low-schooled participants where the impact of a more limited education level seems to be more significant than impact of the lesion. In other words, the education effect was more determining than the lesion effect. In a Brazilian study on neuropsychological assessment of RH brain-damaged participants’ communication,^35^ an effect of this neurological impairment was observed in conversational discourse, verbal fluency and emotional and linguistic prosody tasks. However, this investigation did not analyze the relationship between RH lesion influence and patients’ education. A further analysis, yet to be published, did not detect any lesion effect in semantic judgment, metaphor and indirect speech act interpretation, or linguistic prosody comprehension tasks, probably because the schooling effect was more significant than lesion influence, there being no interaction between these two factors.

On the other hand, despite this evidence of a more prominent influence of the years of schooling on RH lesion, a study which assessed aphasic patient performance on the Montreal-Toulouse 86 Aphasia Battery, comprising naming, oral comprehension and repetition tests, found a schooling effect only in non-brain damaged individuals.^36^ However, aphasics’ performance was not influenced by schooling level. Thus, the findings reported above suggest that the overlap between the sociocultural effect of schooling and the neurological factor of the vascular lesion occurs only in the context of an RH lesion, and is not replicable in LH brain-damaged individuals, a distinction which needs further investigation.

In light of the contradictory results, Reis and Petersson^15^ suggested that, although the literature reports schooling influence in normal individuals, studies with brain-damaged populations have not corroborated the impact of this effect. In research in clinical populations, the schooling variable is a criterion for matching control and neurological groups, in order to favor a more precise analysis of the relationship between pathology and cognitive performance. Research on Alzheimer's disease,^37^ Parkinson disease,^38^ RH brain damage,^39^ among other neurological conditions, illustrates this concern. In fact, the most important aspect to be investigated is not to verify whether education influences neurologically impaired population performance, but rather to analyze to what extent lesion and schooling effects interact with the varying neuropsychological task performance. For instance, in severe cases of Broca’s aphasia, independently of the participant’s schooling level, the naming task, for example, cannot be adopted. The same applies to advanced cases of Alzheimer’s disease in simple tests of digit memory. However, in moderate and mild cases, the complexity of cognitive processing of the task may favor the emergence of the schooling effect. Thus, it is possible that in a task requiring only linguistic processing, disturbance in this function will be more important than the education effect, which develops different cognitive abilities. However, if a task also demands several abilities developed and required at school, such as inferential processing, text comprehension and verbal fluency tasks, it is possible that, besides the lesion effect, the education factor also becomes evident. The complexity of the relationship between schooling and the lesion effect does not detract from the importance of its discussion, since the differences in education and reading habits are very evident in our country and probably in other Latin American countries, thereby demonstrating that this controversial and relevant issue needs further investigation.

### The relation between education and dementia

Since the 1990s, several studies have reported that a higher education and cultural level reduces the impact of dementia^40^. However, the number of studies which have not observed differences in the dementia processes in patients with varying education levels carry equal weight.^41,42,43^ In fact, this issue remains very complex.

Taking studies on schooling influence on dementia processes as a starting point, several models have attempted to explain the impact of this variable on neurocognitive organization. One of these theoretical paradigms is the cognitive reserve hypothesis. This hypothesis emerged when researchers noted there was no direct relationship between lesion severity and clinical symptoms.^44^ Two groups of models of the cognitive reserve paradigm will be briefly addressed:

(1) passive models and
(2) active models.

The passive models conceive the idea of “reserve” as being similar to “hardware”, and therefore consider anatomical variables as an index measure of reserve. Yet the active models compare “reserve” to “software”, reflecting individual differences in terms of the way each person approaches a task, using, for instance, different strategies to tackle it. Active models assume that individual performance is mediated by several factors such as education (considering years of school attendance or higher degree attained), premorbid IQ (tested by the Wechsler Adult Intelligence Scale (WAIS) Vocabulary subtest or the National Adult Reading Test), and profession/occupation experience.^42^

As stated above, researchers noted there was no direct relationship between lesion severity and clinical diagnoses. A first attempt to explain this discrepancy was proposed^45^ based on synapse count or brain volume, which would be related to brain reserve capacity thus characterizing a passive model. The author’s assumption was supported by research showing positive correlation between brain size and cognitive functioning in pathological and healthy populations. A greater brain “reserve” would thus prevent or reduce clinical manifestations. To illustrate, an fMRI study^46^ found positive correlation between brain volume and general intelligence in 97 healthy elderly adults. However, caution must be exercised when interpreting these data, since they may reflect a correlation between age and frontal lobe atrophy in low-schooled individuals.^43^

Another example based on the passive model to explain cognitive reserve, with emphasis on cerebral organization changes, was a PET study in elderly illiterate women.^13^ The study identified cerebral areas developed by learning a writing system. According to the authors, oral or written language utilizes:

(1) areas in the auditory cortex and in the subcortex for phonological modulation;
(2) auditory and multisensory integration cortexes for lexical-semantic components;
(3) visual areas for reading;
(4) parietal cortex and dorsolateral visual area, as well as motor areas, for writing, and
(5) interconnections among all these areas.

The researchers suggested the occurrence of an increase in the amount of connections upon schooling, especially of those which demand visual associations with the RH, including higher participation of the *corpus callosum*. To date, studies run by these authors have confirmed the occurrence of higher activations in the RH in illiterates along with lower activations in posterior regions of the *corpus callosum*, corroborating the underlying assumptions made by the model. However, these studies are recent and warrant more in-depth investigation.

The active model has been corroborated by PET studies in Alzheimer’s disease and healthy individuals.^47,48^ By analyzing individuals’ performance in visual recognition tasks, the researchers revealed a correlation between data on task resolution and participants’ cognitive reserve.

Among the active models, the aspect which has been most investigated is education, which has been assumed to produce greater cognitive resources, crucial to overcome functional losses and to search for compensatory strategies. However, one experiment did not identify cognitive reserves as a determinant for the onset of degenerative diseases caused by aging.^20^ The authors of this experiment postulated that a higher formal education level did not modify the course of Alzheimer’s disease. Instead, low-schooled individuals demonstrated, in studies on autopsies of patients diagnosed as having dementia, a higher tendency for strokes, due to the incidence of minor cerebrovascular accidents.

Evidence from this review of the effect of literacy and formal education on human brain organization suggested that schooling processes influenced cerebral organization of healthy individuals, as well as in brain-damaged individuals and those with dementia. Concerning illiteracy, the systematic pioneering studies developed by André Roch Lecours, as well as the continuity of his investigations, were essential to alert the scientific and clinical communities to take into account cerebral flexibility and educational experience. The differences among the groups with different schooling levels and literacy conditions probably correlate to a higher sensitivity to the education factor whenever cognitive abilities are examined in formal testing settings. According to this assumption, illiterate individuals are less exposed to visual stimuli than those who have experienced educational environments. Experiencing formal education potentially exposes the literate person to frequent linguistic and visuospatial stimulation, familiarizing them with this type of stimuli in contrast to illiterates, who do not frequently experience this.

The variability among low-schooling and illiterate individuals should be investigated further. For instance, the hypothesis that the frequency of reading and writing habits may be related to this variability may reflect that, besides the schooling factor, usually considered in the neuropsychological assessment process, the type and frequency of writing and reading habits should also be observed in comparative and normative studies. Moreover, a more frequent and extensive investigation of the relationship between schooling and brain lesions, as well as of schooling and the rate of occurrence and evolution of dementia, represent relevant issues to be pursued.

Currently there are no doubts that the multiple interactions with the environment lead the brain to function in an adaptive form. A large proportion of environmental influences are a product of culture, created by human beings and expressed mainly by their languages. Thus it becomes clear that, despite the constant confirmation that schooling and/or literacy have an impact on cerebral organization and cortical and intra-hemispheric connections, along with a marked influence on performance in neuropsychological tasks, several questions need to be explored: Which changes underlying the schooling process may justify the differences found? What is the interaction between education and right and left hemisphere brain lesion? Does education guarantee a cognitive reserve that prevents or postpones the dementia in Alzheimer's disease? Are populations with different education levels equally susceptible to neuropsychological rehabilitation? Lastly, the complexity of the factors influencing its theoretical basis make neuropsychological research a constant challenge. This challenge is yet greater in terms of understanding the relationships between formal educational experience and cognitive processing of individuals from countries like Brazil, which are renowned for their broad cultural diversity.
